# Characterization and Accelerated Ageing of UHMWPE Used in Orthopedic Prosthesis by Peroxide

**DOI:** 10.3390/ma2020562

**Published:** 2009-05-13

**Authors:** Magda Rocha, Alexandra Mansur, Herman Mansur

**Affiliations:** Department of Metallurgical and Materials Engineering, Laboratory of Biomaterials and Tissue Engineering, Federal University of Minas Gerais / R. Espírito Santo 35, 30160-030, Belo Horizonte, Brazil; E-Mails: fisio.magda@gmail.com (M.R.); aapiscitelli@uol.com.br (A.M.)

**Keywords:** UHMWPE, polymer degradation, polymer oxidation, accelerated aging, prosthesis

## Abstract

Ultra-high molecular weight polyethylene (UHMWPE) has been the most commonly used bearing material in total joint arthroplasty. Wear and oxidation fatigue resistance of UHMWPE are regarded as two important mechanical properties to extend the longevity of knee prostheses. Though accelerated *in vitro* protocols have been developed to test the relative oxidation resistance of various types of UHMWPE, its mechanism is not accurately understood yet. Thus, in the present study an accelerated ageing of UHMWPE in hydrogen peroxide solution was performed and relative oxidation was extensively characterized by Fourier Transformed Infrared Spectroscopy (FTIR) spectroscopy and the morphological changes were analyzed by Scanning Electron Microscopy (SEM). Different chemical groups of UHMWPE associated with the degradation reaction were monitored for over 120 days in order to evaluate the possible oxidation mechanism(s) which may have occurred. The results have provided strong evidence that the oxidation mechanism is rather complex, and two stages with their own particular first-order kinetics reaction patterns have been clearly identified. Furthermore, hydrogen peroxide has proven to be an efficient oxidative medium to accelerate ageing of UHMWPE.

## 1. Introduction

Ultra-high molecular weight polyethylene (UHMWPE) is a biomaterial widely used as part of prostheses that require articulating surfaces, such as knee endoprostheses, for its excellent mechanical qualities. Approximately one million UHMWPE components are implanted yearly worldwide. Two major problems limit the life of UHMWPE prosthesis - wear and delamination, with both phenomena being mainly the result of chemical oxidation of polymer [1,2,3,4,5,6]. Polyethylene wear debris formation continues to be a primary factor in the reduced longevity of total knee replacements [1,2,3]. Wearing causes the release of generated particulate matter that triggers a macrophage reaction leading to chronic inflammation and osteolysis, while delamination, due to the mechanical stress, macroscopically alters the surfaces [2,6]. It is well known in the field of biomaterials that the surface chemistry plays an important role in the interaction of cells, specifically those cells involved in the inflammatory response. Thus, this aspect must be considered in the applications of UHMWPE [2,3,4].

Oxidized UHMWPE is an inherent state that exists in UHMWPE components used in total joint replacements. As such, studies that investigate the interaction of particles with cells and tissue should consider the effect of this surface chemistry in their models. It potentially influences the structural state of the particles, the nature of leachables, their release rates as well as the kinetics of the degradation reaction, and the manner by which biological elements of the implant environment act on the material. In addition, the converse might also be true since cellular interactions are influenced by the oxidation state of surfaces. Furthermore, soluble degradation products released from the materials may be able to influence cell function, remote from the particles themselves [3,4,5,6]. It has also been well established that these UHMWPE components are susceptible to oxidative degradation through consolidation of the resin at high temperatures and pressures, sterilization by gamma irradiation, storage of the irradiated UHMWPE components and the implantation environment. The oxidation of UHMWPE components has been linked to changes in the mechanical properties of the material, such as decreased fatigue strength and the production of wear particles around the site of the implant [4]. Until the 1980s most studies focused to the hip. Only in the last years some *in vitro* studies reporting chemical oxidation of UHMWPE have focused to the knee and its mechanism still remains unclear. Besides that, maximum oxidation matching criteria have been associated with the chemical and physical properties of UHMWPE, but just a few studies have focused on microstructural changes related to the accelerating aging that actually occurs in UHMWPE prosthesis. Despite the large number of works in recent years that seem to indicate a satisfactory behavior of UHMWPE for bearing applications, further research needs to be carried out in order to properly predict lifetime of these prostheses when implanted [5,6,7,8,9,10,11].

The oxidation of UHMWPE is in reality a complex sequence of various cascading reactions which is not fully understood. Despite the intensive focus on ageing methodologies for UHMWPE over the last few years, there remains much debate over the most effective way of accelerating ageing of this material to simulate both shelf and *in vivo* ageing. The ideal accelerated ageing protocol would simultaneously reproduce the chemical changes in materials, as well as, the depth profiles of these changes, including oxidation. However there are no ageing protocols that have been adequately validated to replicate real-time shelf and *in vivo* ageing of highly cross-linked or conventional PE [9]. Protocols and standards for accelerated ageing of UHMWPE usually involve heating for 21 days at 80 °C in air or heating for 14 days at 70 °C under 5 bar oxygen [12,13]. Though all the aging environments may produce similar levels of surface oxidation, it is clear that the environments produce oxidation profiles that do not otherwise resemble the profile actually developing under shelf aging [12]. Also, interlaboratory studies have identified that, although some materials can be ranked successfully by both these heating methods, there is poor interlaboratory reproducibility [14]. It was also verified that hydrogen peroxide exposure causes more severe oxidation than either air or hyaluronic acid and that unsterilized samples and even those sterilized in ethylene oxide are resistant to oxidation under all conditions, except hydrogen peroxide aging [15].

The aim of this research was to investigate and characterize the accelerated degradation of UHMWPE under exposure to an aggressive hydrogen peroxide medium. The degree of oxidation was chemically evaluated by FTIR spectroscopy and the morphological changes were analyzed by Scanning Electron Microscopy. Moreover, the kinetics and mechanism of the oxidation reaction were investigated for potential use as a protocol for accelerated ageing and materials lifetime prediction.

## 2. Results and Discussion

### 2.1. Characterization of UHMWPE

#### 2.1.1. FTIR Spectroscopy Characterization – Reference UHMWPE

**Figure 1:**
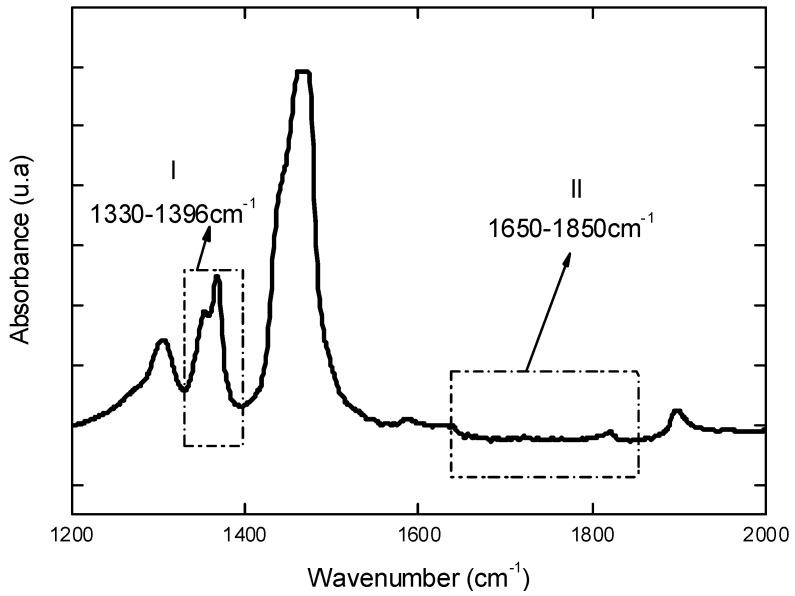
FTIR spectrum of reference UHMWPE in the region from 1,200 to 2,000 cm^-1^ used to estimate the total oxidation index (Iox) according to ISO 5834-4.

Initially, UHMWPE used as reference materials was characterized before undergoing the oxidative procedure. Figure 1 shows the FTIR spectrum in the region between 1,200 and 2,000 cm^-1^ of the UHMWPE which was utilized for calculating the initial Oxidation Index (Iox) according to ISO 5834-4 [5,7]. Briefly, this oxidation factor Iox is assumed as the peak area at 1,650 – 1,850 cm^-1^ divided by the peak area at 1,370 cm^-1^. This gives an overall relative uncertainty of 10%. The results have indicated an average value of Iox = 0.03 (S.D. = 0.01), within the range (0.00 – 0.07) of previously reported studies [9,10].

#### 2.1.2. SEM Characterization – Reference UHMWPE

**Figure 2:**
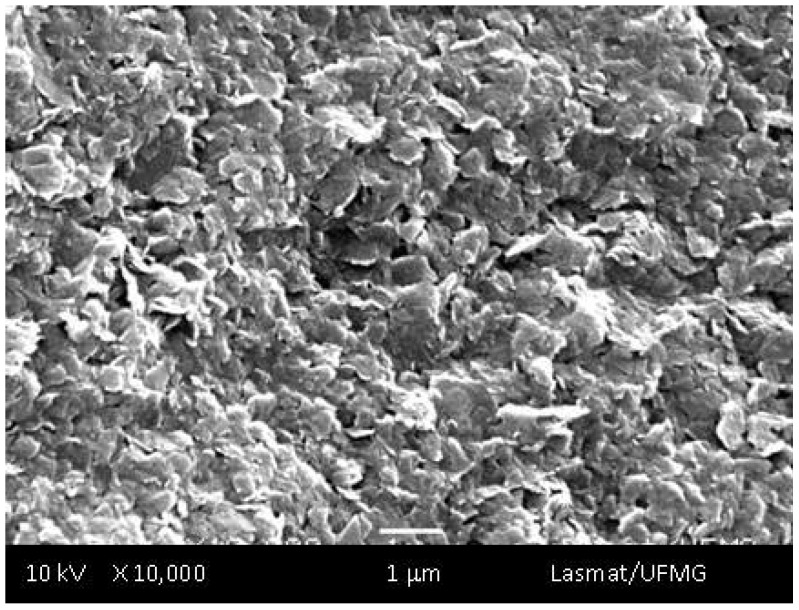
SEM image of UHMWPE surface used as reference prior to oxidation showing a “wave-like” morphological structure.

Figure 2 shows a typical SEM image from the UHMWPE “as supplied” used as the reference sample before the oxidation procedure. The surface morphology is uniform with the microstructure predominantly revealing a “wave-like” shape, which is usually a feature of relatively ductile material capable of large deformation [9]. The EDX spectrum of the UHMWPE samples (not shown) did not detect any chemical elements present as contaminants. This aspect is compatible with UHMWPE of low cross-linking related to sterilization by gamma-irradiation according to the literature [9]. Hence, based on the oxidation index (Iox) and the characterized microstructure of the samples, it is reasonable to assume that the UHMWPE can be used as reference control of the accelerated aging experiments.

### 2.2. Characterization of UHMWPE Submitted to Accelerated Aging by H_2_O_2_

#### 2.2.1. Oxidizer Generation – Background

Despite the intensive focus on ageing methodologies for UHMWPE there remains much debate over the most effective way to accelerate ageing of this material. Many studies have analyzed the changes in UHWMPE after irradiation and natural aging [5,6,8,9,10,11]. However, in-depth analysis in terms of physico-chemical aspects, for instance oxidation index, crystallinity, formation of radicals, reaction kinetics and pathways are not yet totally understood. H_2_O_2_ is one of the most powerful oxidizers known. Also, through catalysis, H_2_O_2_ can be converted into hydroxyl radicals (•OH) with have very high reactivity. Hydrogen peroxide always decomposes spontaneously and exothermically into water and oxygen gas (Equation 1):

H_2_O_2_(aq) → H_2_O(l) + ½O_2_(g) (1)

This process is very thermodynamically favorable (ΔH° = −98.2 kJ mol^−1^; ΔG° = −119.2 kJ mol^−1^; ΔS = 70.5 J mol^−1^ K^−1^). The rate of decomposition is dependent on the temperature and concentration of the peroxide, as well as the pH. Furthermore, the theory supports by a vast body of experimental evidence that aerobic organisms generate powerful pro-oxidant species such as hydrogen peroxide. Consequently, the oxidation by hydrogen peroxide solution was used as an accelerated aging procedure and samples were collected at different time intervals.

#### 2.2.2. FTIR Spectroscopy – Oxidation

FTIR spectroscopy was used for characterizing the oxidized UHMWPE samples (UHMWPE-Ox). In Table 1, some major bands usually associated with oxidative degradation of UHMWPE according to the broadly reported literature [6,7,8,9,10,11,16,17,18,19,20] are presented. The UHMWPE-Ox samples have exhibited some of these absorption bands after being submitted to the aging assays.

**Table 1:** Major FTIR bands associated with UHMWPE and its oxidized species.

Band Region (cm^-1^)	Description	Reference
3,450 – 3,350	Hydroperoxide and alcohol	[6,10,11,19]
1,710 – 1,740	Carbonyl species: ketones, carboxylic acid, aldehydes,	[9,10,11]
1,100 – 1,400	Ethers and other –C-O-C groups	[9,10,11]
800 – 1,000	Unsaturated bonds, trans-vinylene groups	[6,10,11]

**Figure 3:**
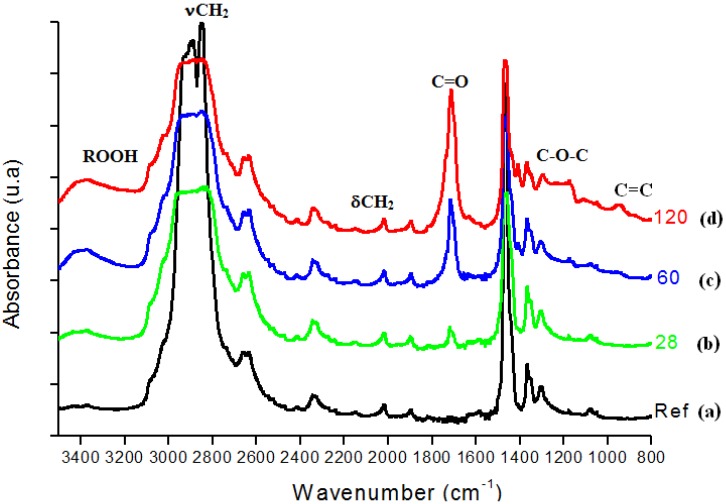
(a) FTIR spectra of reference UHMWPE and UHMWPE oxidized for (b) 28 days, (c) 60 days and (d) 120 days.

**Figure 4:**
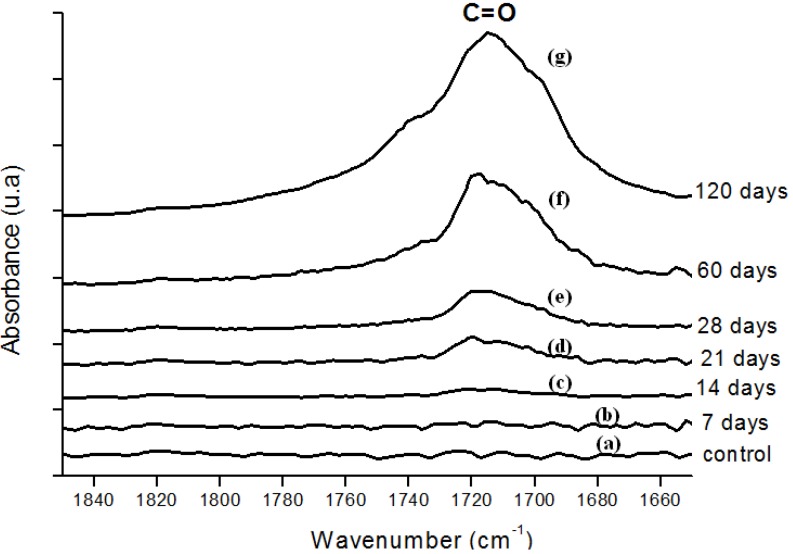
(a) FTIR spectra in the carbonyl (C=O) region of reference UHMWPE and UHMWPE submitted to peroxide accelerated ageing for different periods of time: (b) 7 days; (c) 14 days; (d) 21 days; (e) 28 days; (f) 60 days and (g) 120 days.

In Figure 3, FTIR spectra from UHMWPE before and after 28, 60, 120 days of accelerated aging in hydrogen peroxide are presented. The main changes in the FTIR spectra upon oxidation of polyethylene samples involved the formation of typical products such as isolated hydroperoxides (3,550 cm^-1^), hydrogen bonded hydroxyls including hydroperoxides (3,410 cm^-1^), lactones (1,860 cm^-1^), esters (1,740 cm^-1^), acids and ketones (1,710 – 1,720 cm^-1^). In addition, an increase in the absorbance in the 1,400 – 1,180 cm^-1^ region associated with –C-O-C vibrations and in the region from 800 – 1,100 cm^-1^ mostly related to unsaturated C=C groups was noted. Figure 4 refers to the main peak in the FTIR spectra in the 1,700 – 1,750 cm^-1^ region, corresponding to the strong signal of carbonyl (C=O) groups, which is rather dependent of specimen degradation. A significant absorbance increase was observed in the test period from 7 up to 120 days. The carbonyl functional group is common to several chemical species, for instance ketones, esters, carboxylic acids and lactones, among others. Thus, such results provide strong evidence that UHMWPE oxidation has taken place via chemical reactions of the polyethylene chain with hydrogen peroxide from the aging solution.

**Figure 5:**
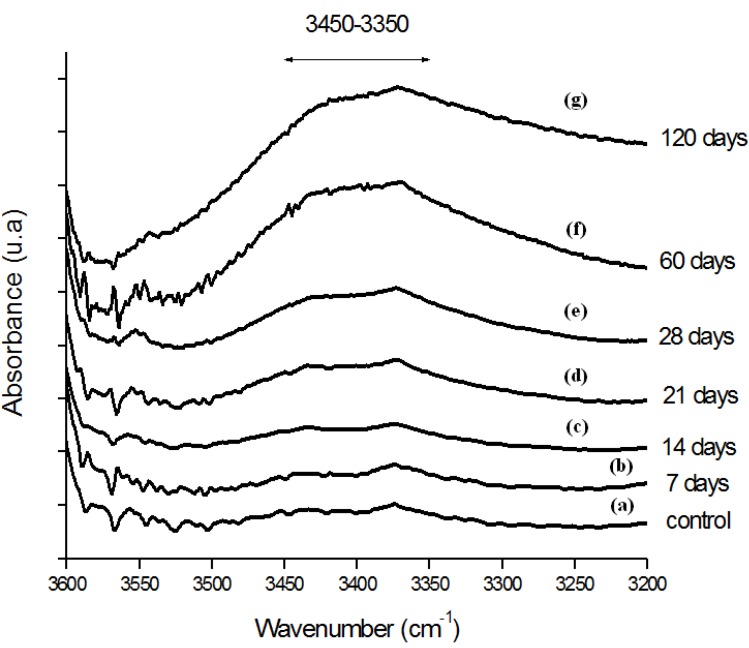
(a) FTIR spectra in the hydroperoxides (ROOH) region of reference UHMWPE and UHMWPE submitted to peroxide accelerated ageing for different period of time (b) 7 days; (c) 14 days; (d) 21 days; (e) 28 days; (f) 60 days and (g) 120 days.

**Figure 6:**
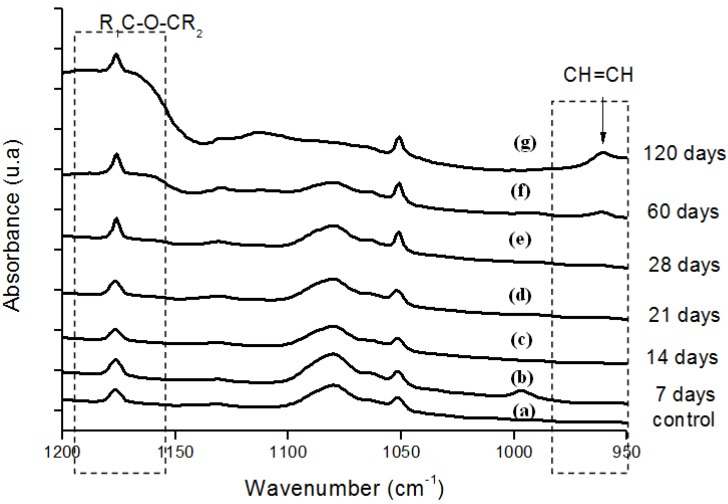
(a) FTIR spectra in the ether (-C-O-C-) and trans-vinylene (-CH=CH-) regions of reference UHMWPE and UHMWPE submitted to peroxide accelerated ageing for different periods of time: (b) 7 days; (c) 14 days; (d) 21 days; (e) 28 days; (f) 60 days and (g) 120 days.

Moreover, the hydroperoxide-specific IR vibrational region also supports these findings, as shown in Figure 5. The FTIR spectra in Figure 6 shows the evolution of the C-O-C and trans-vinylene (CH=CH) bands as the UHMWPE oxidation time is increased up to 120 days. A similar trend was observed for all chemical groups investigated, that means, carbonyl, hydroperoxides and vinylenes species have showed remarkably higher content for longer oxidation time. In general, almost no detectable oxidation could be observed in the first 7-days period. After that, the oxidation has increased considerably in the timeframe analyzed.

**Figure 7:**
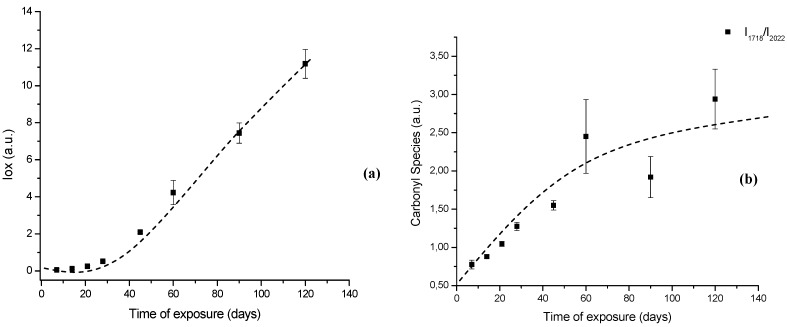
(a) Evolution of UHMWPE degradation in H_2_O_2_ estimated by FTIR using the Oxidation Index (Iox) at different aging times. (b) Specific carbonyl group index (I_C=O_) related to polymer oxidation up to 120 days.

In order to perform a more in-depth investigation of the UHMWPE degradation as far as quantitative analysis was concerned, the spectral regions recommended by ISO 5834-4 (2005) were used to estimate the oxidation degree. The total oxidation index (Iox) was calculated as described in the *“Experimental Section”* (Equation 5). Also, the specific carbonyls region was monitored and another parameter calculated according to Equation 6 in the *“Experimental Section”* (I_C=O_). The I_C=O_ is mainly due to the presence of ketones estimated from absorbance ratio (as peak heights) between the C=O stretching (νC=O) component of ketones at 1,710 – 1,720 cm^-1^ and the band at 2,022 cm^-1^ from the CH_2_ bending vibrations (internal standard, δCH_2_, “twist” bend ) which has been reported in the literature [7,8,9,10,11]. The results with data from 7, 14, 28, 45, 60 and 120 days test periods are shown in Figures 7a,b. The curve 7a, based on the overall reaction (Iox), clearly indicated that oxidation of the UHMWPE has taken place, with a slow evolution up to approximately 28 days. Then, a steep rise could be observed up to 120 days of evaluation. Those oxidation index (Iox) values are supported by reported research on retrieved prosthesis after degradation and severe inflammation processes under patient use. On the other hand, curve 7b related to specific carbonyls (I_C=O_) monitored by FTIR has indicated a steady and constant increase on concentration from the early beginning until approximately 45 days, then followed by a gradual reduction up to 120 days. Hence, FTIR has proven to be an important tool, not only for investigating UHMWPE degradation, but also providing relevant information concerning reaction kinetics and mechanism.

#### 2.2.3. Kinetics and Mechanism of UHMWPE Oxidation by FTIR Spectroscopy

The reaction kinetics was investigated in order to obtain a better understanding of the oxidation of UHMWPE. It should be pointed out that a complete investigation of kinetics and mechanism is beyond the scope of the present research. Nevertheless, a preliminary analysis is proposed, aiming to address the long term performance of UHMWPE under peroxide activity. The essential assumption relies on first order reactions. Hence, it would be modeled as Equations 2 and 3:

(2) $$\text{Differential form: }Rate=\frac{dA}{dt}=k[A]$$

Natural Log form: ln([A]/[A]_0_) = kt (3)

where k is a temperature-dependent rate constant; t is the reaction time; [A]_0_ is the initial concentration of oxidized specie; [A] is the instantaneous concentration of oxidized specie at any time.

**Figure 8:**
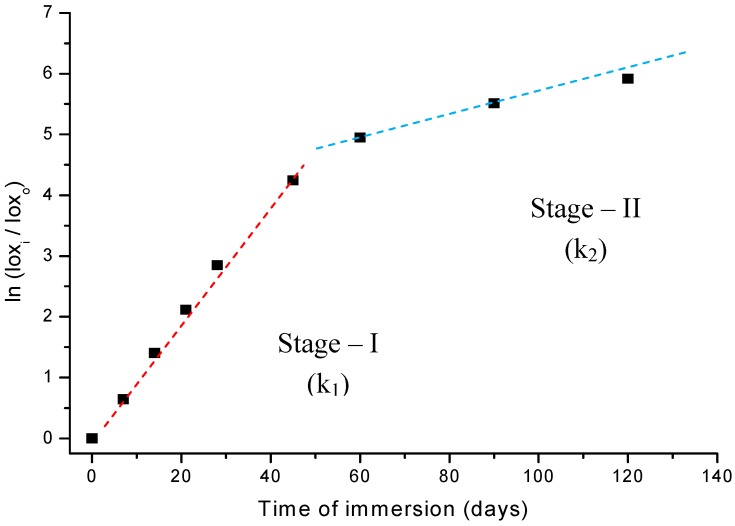
Study of reaction kinetics associated with the oxidation of UHMWPE in H_2_O_2_.

Thus, if the UHMWPE degradation via an oxidation reaction in peroxide follows first-order kinetics, a plot of ln (A/A_0_) against time will be linear, with slope k as the reaction constant parameter. Indeed, the results shown in Figure 8 fit the expected behavior remarkably, when the whole oxidation curve is interpreted as a two-stages pattern. In other words, it should be analyzed as two separate first-order reactions, with two different slopes. The linear regression from the first-stage up to 45-days (stage-I) has given a very good curve fitting to the data points with parameters R^2^ = 0.99 and k_1_ = 1.11E^-5^ s^-1^. The second-stage (stage-II) values were R^2^ = 0.98 and k_2_ = 1.86E^-6^ s^-1^, from 60 to 120 days of reaction. As the peroxide concentration in solution and temperature (37 ºC) were maintained unaltered during the whole experiments, it is reasonable to attribute such strong change in the reaction rate (of about 600%) to a variation in the oxidation mechanism on passing from stage-I to stage-II. Therefore, they must have quite different activation energy (Ea) as showed by Arrhenius Equation (equation 4):

k = k_0_e ^-Ea/RT^ (4)

As k_1_ ≅ 6. k_2_, thus, Ea_1_ < Ea_2_. That means, the activation energy at the first stage (Ea_1_) is lower than that at longer ages (Ea_2_, stage-II). The difference can be estimated from (4) where: R = 8.31451 J mol^-1^ K^-1^, T=335 K, Δea = RT ln(k_1_/ k_2_) and ΔEa= (Ea_1_ – Ea_2_) = -5.0 kJ mol^-1^

These results are supported by assuming a very short induction period related to the initial decomposition of hydrogen peroxide and the formation of hydroperoxy radicals. Because of the very high initial concentration of hydrogen peroxide (~ 8.8 mol L^-1^) its decomposition into radicals (•OH) is expected to be rather fast. Then, the polymer chain would be readily attacked and broken at the surface by these just formed radicals leading to the formation of hydroperoxides. In addition, the surface energy is always favorable to reactions due to defects, incomplete bonds, higher mobility of atoms at the liquid-solid interface. Such hypotheses have been reported by other authors such as in the studies of the oxidative degradation mechanism of polyethylene material by Gugumus [16]. Despite the absence of a consensus about the UHMWPE degradation mechanism there is full agreement that the oxidation of polyolefins involves the formation and decomposition of hydroperoxides as metastable species. These hydroperoxide intermediates may undergo cascading reaction routes causing the appearance of several sub-products such as alcohols, aldehydes, carboxylic acids, ketones, esters, ethers, unsaturated bonds such as trans-vinylene or cyclic species like gamma-lactones [5,6,10,11,16,17,18,19,20,21]. That would be the overall trend for stage-I in the oxidation pattern of UHMWPE. Then, as the UHMWPE is damaged by the growing number of oxidized species, at the surface and near-surface depth, the original organized structure of polymer chain is exponentially disordered, the degraded layer is thicker, causing a drastically decrease on the oxidation rate as verified in stage-II. Associated with that, some polymer chain crosslinking might be expected and the restricted diffusion of oxygen and oxidizing radicals through the high dense and linear UHMWPE network would most likely cause the oxidation kinetic rate to sharply drop. Our results are consistent with the literature [3,8,12,16,17,18,19,20,21,22,23] which has shown that there is a distinct threshold of oxidation index values that must be exceeded in order to observe important alterations at the concentration of oxidized species generated in UHMWPE materials. Deeper into the UHMWPE there will be less oxygen available for reaction, so most of the radicals will combine to produce cross-links. In summary, in stage-I the kinetic mechanism is mostly controlled by surface reactions and in stage-II it is mainly determined by “bulk” (volume) reactions, as supported by k_1_ > k_2_ and ΔEa < 0 or Ea_1_ < Ea_2_.

#### 2.2.4. Morphology and Structure UHMWPE Oxidation by SEM

**Figure 9:**
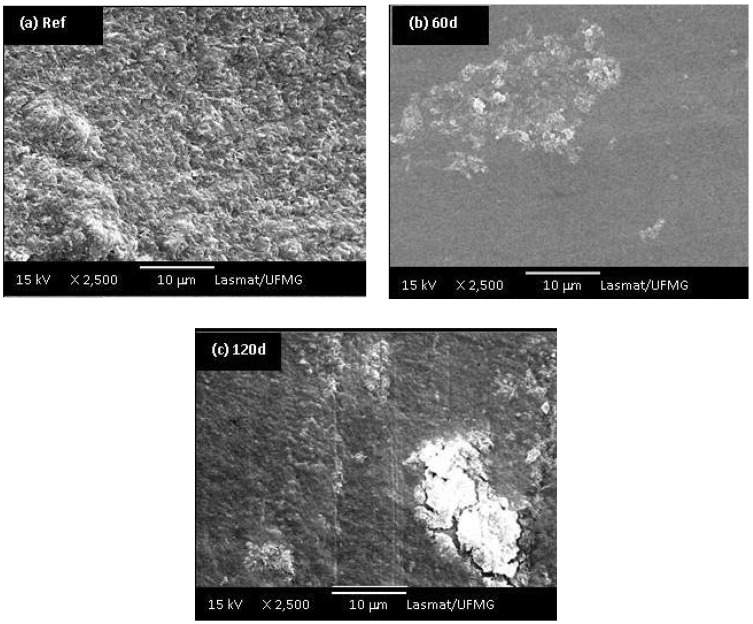
(a) SEM images of UHMWPE reference sample; UHMWPE oxidized with H_2_O_2_ for (b) 60 days and (c) 120 days (at 2,500 X magnifications).

Morphological differences among the UHMWPE samples before and after 60 and 120 days of oxidation in hydrogen peroxide solution can be observed in the SEM images presented in Figures 9a,b,c. It is evident that a transition from a surface with random roughness (Figure 9a, before oxidation) to a smoother surface while increasing the accelerated aging time of oxidation (Figure 9b,c) has taken place. In addition, the samples oxidized for 60 and 90 days showed delamination, pitting and cracks on their surface (Figures 9b,c). Medel and co-workers [8] pointed out that despite the oxidative reaction the degradation is mostly associated with the increase on levels of pitting and delamination, which will cause premature failure of the components. Thus, the oxidation of UHMWPE is a rather complex system justifying the intense research in the field in the last 2 – 3 decades. Despite the abundant literature, it is yet not fully understood how much the “*in vivo*” degradation will depend not only on the material composition and processing, but if it is a multifactorial result from the patient metabolism, gender, age, dynamic behavior and activities, surgical procedure and so forth.

Based on the FTIR and SEM results, it was verified that the changes in UHMWPE induced by H_2_O_2_ ambient reasonably resemble the chemical species and the morphological and structural alterations caused by shelf and *in vivo* oxidation. Despite the fact that H_2_O_2_ is more severe with regards to the oxidative potential than the conditions usually used for the heating protocols, this approach has a significant advantage because it can successfully oxidize unsterilized UHMWPE, which cannot be done under other reported acceleration conditions. Hence, these findings open up interesting alternatives whereby such a method can be used to compare and rank the oxidation response of different resin types, sterilization methods, environment during sterilization and packaging, process support, and thermal stabilization. Also, it may be a useful tool on monitoring and controlling the quality of UHMWPE related to common variations in the manufacturing process. Moreover, the accelerated aging procedure based on H_2_O_2_ would be a feasible choice for investigating the behavior of novel UHMWPE-derived materials with incorporated anti-oxidant agents like α-tocoferol and others.

## 3. Experimental Section

### 3.1. Materials

Experiments were conducted with ram-extruded UHMWPE bar stock GUR 1020, commercial grade approved for surgical implants (ISO 5834-2/2005), kindly donated by Ticona Engineering Polymer (USA). The typical properties of UHMWPE as provided by the supplier are listed in Table 2. It is important to emphasize that all samples were prepared from non-sterilized UHMWPE based on the reported literature that gamma-radiation sterilization procedure leads to the formation of polymer chain crosslinking, peroxides, alkyl radicals and unsaturated bonds. Even with some thermal annealing the post-sterilized UHMWPE would have the original structure changed. Furthermore, despite the fact that studies suggest that sterilization using ethylene oxide (EtO) gas does not substantially influence the physical and chemical properties of UHMWPE others have found evidence of surface residues. Therefore, it was the choice from authors to investigate the actual kinetics of UHMWPE degradation under accelerated conditions based upon the polymer original or virgin structure.

**Table 2:** Material properties of UHMWPE bar stock based on data sheet from manufacturer.

UHMWPE	
Resin type	GUR 1020
Manufacturer	Ticona
Manufacturing Method	Ram extrusion
Fabricated Form	Annealed
Average molecular wt (molar mass) [g/mol × 10^6^]	5.166 to 5.415
Crystallinity; DSC, (20 °C –160 °C) [%]	66 – 71
Density [Kg/m^3^]	934
Tensile stress at yield (tensile strength) [MPa]	> 23
Tensile stress at break [ultimate tensile strength [MPa]	> 52
Elongation at break [%]	> 460
Young’s modulus [MPa]	> 575
Melting Point DSC, 10K/min [ºC]	137.5
Glass Transition Temperature Tg [ºC]	-110
Surface and bulk Oxidation Index; material shelf aged 1 year in air (ASTM F2101-01)	0.00

### 3.2. Sample Preparation

The bar stock was sectioned with a steel blade saw into 5 cm × 5 cm × 5 cm blocks and then samples were sliced with thickness from 150 to 250 µm. All the samples were cleaned in ethanol (P.A., Sigma) and distilled water ultrasound bath, dried in air and submitted to experimental analysis. Non-oxidized slices triplicates (n = 3) of UHMWPE (“reference control group”) were immersed in 30 mL of hydrogen peroxide and incubated at 37 ºC. The oxidant solution consisted of 30 v/v% hydrogen peroxide (analytical grade, H_2_O_2_, > 30 v/v%, ~ 8.8 mol L^-1^), which was replaced every five days, in order to maintain the activity of the solution. This interval was chosen based on the half-life of H_2_O_2_ at 37 ºC to be about seven days. The experimental aging times were monitored at 0, 7, 14, 21, 28, 60, and 120 days and such oxidized samples were denominated UHMWPE-Ox.

### 3.3. Characterization

Fourier Transformed Infrared Spectroscopy (FTIR) spectra were collected in transmission mode with wavenumber ranging from 4,000 to 400 cm^-1^ during 64 scans, with 2 cm^-1^ resolution (Paragon 1,000, Perkin-Elmer, USA). The FTIR spectra were normalized and major vibration bands were identified and associated with the main chemical groups. The total level of oxidation (Iox) was determined by FTIR according to ISO 5834-2 [7] as showed in Equation 5 where A_O_ = Integrated area from 1,650 cm^-1^ to 1,850 cm^-1^ and A_R_ = Integrated area from 1,330 cm^-1^ to 1,396 cm^-1^. The areas were calculated through Origin Program®, version 7.0. Regarding to the evaluation of oxidation, the ISO 5834 is similar to ASTM Standard F648, whereas the oxidation index standard (Part 4) is the same method as ASTM F2102:

I_Ox_ = A_O_/A_R_ (5)

Moreover, FTIR was used to investigate the oxidation mechanism of UHMWPE whereby specific bands of different chemical species were monitored [6,7,9,10,11], as shown in Equation 6 for the index based on carbonyl compounds (-C=O):

I_C=O_ = I_1714_/I_2022_ (6)

In this equation I_1714_ is the peak height related to carbonyl and I_2022_ is the peak height related to reference band δCH_2_.

Scanning Electron Microscopy coupled to EDX microprobe (SEM/EDX) technique was used to assess the morphology and microstructure changes due to sample oxidation (JSM 6,360LV, Jeol/Noran). Before SEM analysis, the samples were sprayed with an ultrafine layer of gold using a low deposition rate, refrigerated and placed at the maximum distance from the target to prevent damaging them. Images of secondary electrons (SE) were obtained using an accelerating voltage of 10 kV – 15 kV.

## 4. Conclusions

In summary, this study has showed the usefulness of an accelerated aging procedure based on hydrogen peroxide for investigating the chemical stability of UHMWPE. It has focused on the characterization of UHMWPE samples that have been immersed for different periods of time reaching up to 120 days. FTIR spectroscopy has proven to be an important tool for evaluating UHMWPE degradation as major species directly associated with oxidation reactions, for instance hydroperoxides, ketones, esters and vinylenes, were identified. Also, relevant and new information concerning the reaction kinetics and mechanism are presented, with two main stages being verified. Briefly, in stage-I the kinetics mechanism is mostly controlled by surface reactions and in stage-II, they are mainly determined by “bulk” (volume) reactions, as supported by k_1_> k_2_ and Ea_1_ < Ea_2_. The mechanism of oxidation of UHMWPE is largely unknown and still needs to be more in-depth investigation in order to minimize or ideally prevent it from occurring under clinical usage in orthopedic implants. This preliminary research has moved a step further on a better understanding that complex system and it has indicated hydrogen peroxide as an efficient medium to accelerated ageing of UHMWPE which may be used as an important tool on comparing several variables and parameters involved in the research and manufacturing process of UHMWPE.

## References

[1] Desjardins J.D., Burnikel B., Laberge M. (2008). UHMWPE wear against roughened oxidized zirconium and CoCr femoral knee components during force-controlled simulation. Wear.

[2] Renó F., Cannas M. (2006). UHMWPE and vitamin E bioactivity: An emerging perspective. Biomaterials.

[3] Lee A.W., Santerre P.J., Boynton E. (2000). Analysis of released products from oxidized ultra-high molecular weight polyethylene incubated with hydrogen peroxide and salt solutions. Biomaterials.

[4] Sawae Y., Yamamoto A., Murakami T. (2008). Influence of protein and lipid concentration of the test lubricant on the wear of ultra high molecular weight polyethylene. Tribol. Int..

[5] Medhekar V., Thompson R.W., Wang A., Grant McGimpsey W. (2003). Modeling the oxidative degradation of ultra-high molecular-weight polyethylene. J. Appl. Polym. Sci..

[6] Taddei P., Affatato S., Fagnano C., Toni A. (2006). Oxidation in ultrahigh molecular weight polyethylene and cross-linked polyethylene acetabular cups tested against roughened femoral heads in a hip joint simulator. Biomacromolecules.

[B7-materials-02-00562] 7.Implants for surgery -- Ultra-high-molecular-weight polyethylene -- Part 4: Oxidation index measurement method. ISO 5834-4:2005, 2005.

[8] Medel J., García-Alvarez F., Gómez-Barrena E., Puertolas J.A. (2005). Microstructure change of extruded ultra high molecular weight polyethylene after gamma irradiation and shelf-aging. Polym. Degrad. Stab..

[9] Wille B.M., Bloebaum R.D., Ashrafi S., Dearden C., Steffensen T., Hofmann A.A. (2006). Oxidative degradation in highly cross-linked and conventional polyethylene after 2 years of real-time shell aging. Biomaterials.

[10] Medhekar V.S. (2001). *Modeling and simulation of oxidative degradation of ultra-high molecular weight* *polyethylene (UHMWPE)*. Dissertation Degree of Master of Science in Chemical Engineering.

[11] Billingham N.C., Grigg M.N. (2004). The kinetic order of decomposition of polymer hydroperoxides assessed by chemiluminescence. Polym. Degrad. Stab..

[12] Toohey K.S., Blanchet T.A., Heckelman D.D. (2003). Effect of accelerated aging conditions on resultant depth-dependent oxidation and wear resistance of UHMWPE joint replacement bearing materials. Wear.

[13] Buchanan F. (2001). Accelerated ageing and characterisation of UHMWPE used in orthopaedic implants. AZo J. Mater. Online.

[14] Kurtz S.M., Muratoglu O.K., Buchanan F., Currier B., Gsell R., Greer K., Gualtieri G., Johnson R., Schaffner S., Sevo K., Spiegelberg S., Shen F.W., Yau S.S. (2001). Interlaboratory reproducibility of standard accelerated aging methods for oxidation of UHMWPE. Biomaterials.

[15] Goldman M, Lee M., Gronsky R., Pruitt L. (1998). Oxidation of ultrahigh molecular weight polyethylene characterized by Fourier Transform Infrared Spectrometry. J. Biomed. Mater. Res..

[16] Gugumus F. (1996). Thermooxidative degradation of polyolefins in the solid state: Part 1. Experimental kinetics of functional group formation. Polym. Degrad. Stab..

[17] Pruitt L.A. (2005). Deformation, yielding, fracture and fatigue behavior of conventional and highly cross-linked ultra high molecular weight polyethylene. Biomaterials.

[18] Kurtz M.S., Pruitt L.A, Jewett C.W., Crawford R.P., Crane D.J., Edidin A.A. (1998). The yielding, plastic flow, and fracture behavior of ultra-high molecular weight polyethylene used in total joint replacements. Biomaterials.

[19] Bracco P., Brunella V., Zanetti M., Costa L., Luda M.P. (2007). Stabilisation of ultra-high molecular weight polyethylene with vitamin E. Polym. Degrad. Stab..

[20] Costa L., Luda M.P., Trossarelli L., Brach del Prever E.M., Crova M., Gallinaro P. (1998). Oxidation in orthopedic UHMWPE sterilized by gamma-radiation and ethylene oxide. Biomaterials.

[21] Rimnac C.M., Kurtz S.M. (2005). Ionizing radiation and orthopaedic prostheses. Nucl. Instrum. Methods Phys. Res. Sect. B.

[22] Reggiani M., Tinti A., Visentin M., Stea S., Erani P., Fagnano C. (2007). Vibrational spectroscopy study of the oxidation of Hylamer UHMWPE explanted acetabular cups sterilized differently. J. Mol. Struct..

[23] Taddei P., Affatato S., Fagnano C., Bordini B., Tinti A., Toni A. (2002). Vibrational spectroscopy of ultra-high molecular weight polyethylene hip prostheses: influence of the sterilization method on crystallinity and surface oxidation. J. Mol. Struct..

